# Manufacture of IRDye800CW-coupled Fe_3_O_4 _nanoparticles and their applications in cell labeling and *in vivo *imaging

**DOI:** 10.1186/1477-3155-8-25

**Published:** 2010-10-29

**Authors:** Yong Hou, Yingxun Liu, Zhongping Chen, Ning Gu, Jinke Wang

**Affiliations:** 1 State key Laboratory of Bioelectronics, Southeast University, Nanjing 210096, China; 2 Experimental Center of Biotechnology and Biomaterials, BME, Southeast University, Nanjing 210096, China

## Abstract

**Background:**

In recent years, near-infrared fluorescence (NIRF)-labeled iron nanoparticles have been synthesized and applied in a number of applications, including the labeling of human cells for monitoring the engraftment process, imaging tumors, sensoring the *in vivo *molecular environment surrounding nanoparticles and tracing their *in vivo *biodistribution. These studies demonstrate that NIRF-labeled iron nanoparticles provide an efficient probe for cell labeling. Furthermore, the *in vivo *imaging studies show excellent performance of the NIR fluorophores. However, there is a limited selection of NIRF-labeled iron nanoparticles with an optimal wavelength for imaging around 800 nm, where tissue autofluorescence is minimal. Therefore, it is necessary to develop additional alternative NIRF-labeled iron nanoparticles for application in this area.

**Results:**

This study manufactured 12-nm DMSA-coated Fe_3_O_4 _nanoparticles labeled with a near-infrared fluorophore, IRDye800CW (excitation/emission, 774/789 nm), to investigate their applicability in cell labeling and *in vivo *imaging. The mouse macrophage RAW264.7 was labeled with IRDye800CW-labeled Fe_3_O_4 _nanoparticles at concentrations of 20, 30, 40, 50, 60, 80 and 100 μg/ml for 24 h. The results revealed that the cells were efficiently labeled by the nanoparticles, without any significant effect on cell viability. The nanoparticles were injected into the mouse via the tail vein, at dosages of 2 or 5 mg/kg body weight, and the mouse was discontinuously imaged for 24 h. The results demonstrated that the nanoparticles gradually accumulated in liver and kidney regions following injection, reaching maximum concentrations at 6 h post-injection, following which they were gradually removed from these regions. After tracing the nanoparticles throughout the body it was revealed that they mainly distributed in three organs, the liver, spleen and kidney. Real-time live-body imaging effectively reported the dynamic process of the biodistribution and clearance of the nanoparticles *in vivo*.

**Conclusion:**

IRDye800CW-labeled Fe_3_O_4 _nanoparticles provide an effective probe for cell-labeling and *in vivo *imaging.

## Background

In the past decade, the synthesis of iron-based magnetic nanoparticles has rapidly developed for fundamental biomedical applications, including bioseparation [1,2], MRI contrast enhancement [3,4], hyperthermia [5,6], and drug delivery [7,8]. For example, the Fe_3_O_4 _nanoparticle has attracted great attentions for its potential theranostic applications [9-12]. As iron nanoparticles are administered to living subjects in most of their clinical applications, their *in vivo *biodistribution, clearance and biocompatibility must be determined for safe clinical usage. As such, *in vivo *studies of iron nanoparticles have made great progress in recent years.

*In vivo *studies of iron nanoparticles have mainly been performed using magnetic resonance imaging (MRI) [13-18]. MRI is the most widely used technique for imaging magnetic nanoparticles in small animals and humans. A major advantage of MRI is that it can be used to perform real-time imaging of the dynamic biodistribution and clearance of magnetic nanoparticles *in vivo*. However, MRI is still prohibitive to the common research laboratory. Therefore, fluorescence imaging techniques have been developed and applied in studies of magnetic nanoparticles. Iron nanoparticles have been labeled with fluorophores, such as FITC [19-21], rhodamine B [22,23] and rhodamine 6G [18], resulting in the generation of bifunctional labeled nanoparticles, having both MRI and fluorescence imaging functions [24,25]. Magnetic nanoparticles labeled with these conventional fluorophores (350-700 nm absorbing) have often been used to investigate the intracellular distribution of magnetic nanoparticles in cells [17,18,26]; however, these nanoparticles cannot be applied to *in vivo *imaging as the autofluorescence of tissues produce high background under excitation wavelengths less than 700 nm.

In recent years, near-infrared fluorescence (NIRF) imaging technology has been developed and progressively used to obtain biological functions of specific targets *in vitro *and in small animals [27-29]. NIR fluorophores work in the spectrum of 700 to 900 nm, which has a low absorption by tissue chromophores [30]. Therefore, NIRF imaging has minimal background interference. NIR fluorophores also have wide dynamic range and sensitivity, allowing NIRF imaging to obtain detectable signal intensity through several centimeters of tissue [31-33]. Based on these features, NIRF imaging has already been used to label nanoparticles and study their biodistribution, clearance and biocompatibility for *in vivo *biomedical applications. In a recent study, silica nanoparticles were labeled with DY776 and applied for *in vivo *bioimaging, biodistribution, clearance and toxicity analyses [34]. Furthermore, indocyanine green (ICG)-labeled calcium phosphate nanoparticles have been applied for imaging human breast cancer *in vivo *[35].

NIRF imaging has also been applied for the labeling of iron nanoparticles. Maxwell *et al*., used dextran-coated iron oxide nanoparticles (Feridex), covalently modified with Alexa Fluor 750, to label human hepatic stellate cells to monitor the engraftment process *in vivo *[36]. Furthermore, VivoTag 680-conjugated iron oxide particles have been intravenously injected into mice for imaging tumors [37]. Iron nanoparticles, labeled with Cy5.5 (excitation/emission (ex/em), 660/710 nm), have also been used as a MR contrast agent (CLIO) for sensoring the *in vivo *molecular environment surrounding the nanoparticles and tracing the *in vivo *biodistribution of CLIO in liver, spleen and kidneys [38]. Obviously, due to the excellent *in vivo *imaging performance of the NIR fluorophores, the NIRF-labeled iron nanoparticles provide a fine probe for the labeling of biomolecules or cells and *in vivo *imaging [39-42]. However, there is still a limited selection of available iron nanoparticles labeled with NIRF dyes with an optimal wavelength for imaging in the region of 800 nm, where tissue autofluorescence is minimal. Therefore, it is necessary to develop additional alternative NIRF-labeled iron nanoparticles in this area.

This study manufactured water-soluble 12-nm Fe_3_O_4 _nanoparticles labeled with a new NIRF dye, IRDye800CW (Li-Cor Biosciences), which absorb and emit in higher wavelength light (ex/em, 774/789 nm), and investigated their applicability in cell labeling and *in vivo *imaging.

## Results and discussions

### Preparation of IRDy800CW-MNPs

**Figure 1:**
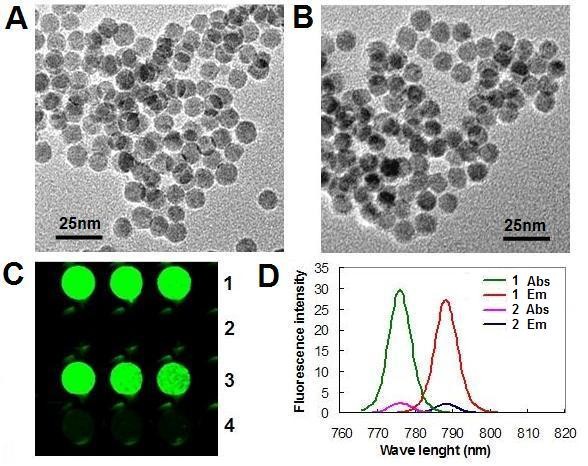
**Characterization of IRDy800CW-MNPs**. (A) TEM image of MNPs. (B) TEM image of IRDy800CW-MNPs. (C) NIRF signal of nanoparticles. (D) Fluorescent spectrum of the nanoparticles. 1: IRDy800CW-MNPs; 2: MNPs. 3-4: The resuspended precipitate and supernatant of the IRDy800CW-MNPs solution after heat treatment and centrifugation. Abs: absorbance. Em: emission.

*M*-2, 3-dimercaptosuccinic acid (DMSA) has often been used as a coating on nanoparticles to improve their water solubility [43-46]. DMSA-coated nanoparticles have abundant carboxyls on their surface [47-49], which can be used to label nanoparticles with fluorophores [23]. Using these features of DMSA, we fabricated novel nanoparticles by firstly creating water-soluble DMSA-coated Fe_3_O_4 _nanoparticles (MNPs), which were then reacted with ethyl-3, (3-di-methylaminopropyl carbodiimide) hydrochloride (EDC) to activate the surface carboxyl groups, following which we covalently crosslinked the NIRF dye, IRDy800CW, to the surface of the MNPs. The monodispersibility and size uniformity of MNPs and the IRDy800CW-labeled Fe_3_O_4 _nanoparticles (IRDy800CW-MNPs) in their prepared water solution were analyzed by TEM. The results demonstrated that both nanoparticles had fine monodispersibility (Figure 1A and 1B). The average size of the nanoparticles was 11.0 ± 1.25 nm in diameter.

The labeling effect of MNPs was evaluated by detecting the NIRF signal of the IRDy800CW-MNPs. In comparison with unlabeled MNPs, the IRDy800CW-MNPs had an intense NIRF signal (Figure 1C). The excitation and emission profiles indicated a peak excitation/emission wavelength of the IRDye800CW-MNPs at 775/788 nm. The Stokes shift for the IRDye800CW-MNPs was 13 nm (Figure 1D). The covalent linkage between IRDy800CW and the MNPs was confirmed by a heating experiment (see Methods), in which the IRDy800CW-MNPs retained the NIRF signal after heat treatment (Figure 1C). If the IRDy800CW was nonspecifically absorbed on the surface of MNPs, the heating treatment would destroy this, resulting in the NIRF signal being found in the supernatant. This result accords with the molecular mechanism that EDC is a carboxyl and amine-reactive cross-linker, which creates an amide bond between carboxyl and amino groups [50].

In this study, the DMSA coating was important for the water solubility and NIRF labeling of the MNPs. Normally, the uncoated iron oxide nanoparticle has a very low solubility due to its hydrophobic surface [51,52]. The DMSA coating makes the surface hydrophilic and dispersible in water solutions [47-49,53-59]. Furthermore, this coating can also improve the biocompatability of iron oxide nanoparticles. In a recent study, DMSA-coated Fe_2_O_3 _nanoparticles were shown to have a low cytotoxicity [57], and have been used to label a variety of mammalian cells [47-49,55]. Conversely, DMSA-coated iron nanoparticles have abundant carboxyl groups on their surface, which is useful for the covalent labeling of nanoparticles by fluorescent dyes [23].

In this study, MNPs were labeled with a newly developed NIRF dye, IRDye800CW, which has several advantages. Firstly, IRDye800CW is a reactive dye [60], which can be easily conjugated to MNPs. This labeling approach can be generalized to other DMSA-coated nanoparticles. Secondly, the excitation and emission of IRDye800CW are in the spectral region where tissue absorption, autofluorescence, and scattering are minimal (800 nm), allowing for the highest signal-to-noise ratio to be achieved in tissue imaging with this dye. For example, IRDye800 absorbs and emits at a higher wavelength light (ex/em, 774/804 nm) than Cy5 (ex/em, 646/664 nm) and therefore produced images with less background resulting from tissue autofluorescence [61]. A comparison of the *in vivo *fluorescent imaging performance of the epidermal growth factor (EGF)-conjugated Cy5.5 (ex/em, 660/710 nm) and IRDye800CW (ex/em, 785/830 nm) revealed that the EGF-IRDye800CW had a significantly reduced background, with an enhanced the tumor-to-background ratio (TBR) in comparison to EGF-Cy5.5 [62]. Thirdly, this dye is highly water-soluble and shows very low nonspecific binding to cellular components, while yielding a very high signal [60,63]. Fourthly, the animal toxicity studies revealed that a single intravenous administration of IRDye800CW carboxylate, at doses of 1, 5, and 20 mg/kg, produced no pathological evidence of toxicity [60]. Furthermore, the animal studies revealed that IRDye800CW and its conjugates were capable of fine *in vivo *imaging in small animal models, such as the mouse [63-67]. IRDye800CW is also reported to be over 50 times brighter than ICG [68]. Based on these features, it is worth developing IRDye800CW-labeled iron nanoparticles as *in vivo *imaging probes with high signal-to-noise ratios.

### Cell labeling with IRDy800CW-MNPs

**Figure 2:**
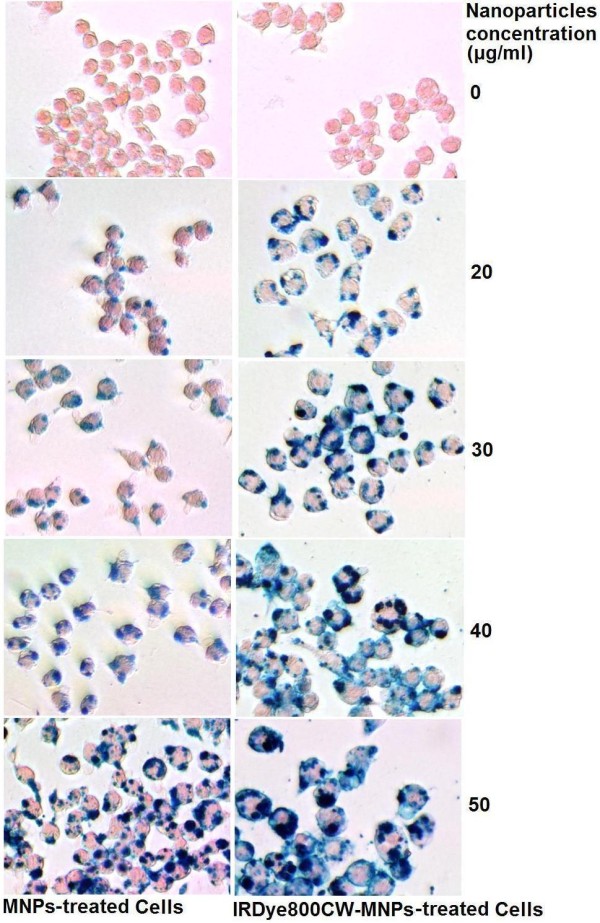
**Prussian blue staining of cells**. The agglomerates of Fe_3_O_4 _nanoparticles are stained in blue.

**Figure 3:**
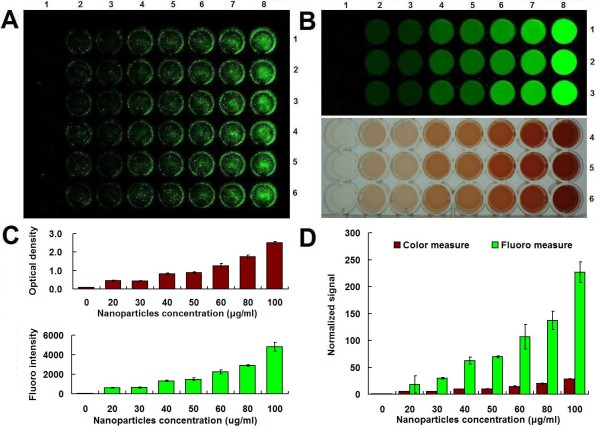
**Measurement of the relative iron-loading of cells**. (A) NIRF signal of cells labeled with IRDy800CW-MNPs at doses of 0, 20, 30, 40, 50, 60, 80 and 100 μg/ml (Column 1-8). Each dose contained 6 repeats (Row 1-6). Cells were washed with PBS before imaging. (B) Measurement of the relative iron-loading of cells (A) with colorimetric and NIRF approaches. Row 1-3: NIRF signals; Row 4-6: Colorimetric signals. (C) The intensity of colorimetric and NIRF signals (B). (D) The normalized intensity of colorimetric and NIRF signals (C). The signal of the nanoparticle-labeled cells was normalized to that of the negative control cell. The error bars represent mean and standard deviations of experiments performed in triplicate.

Cell labeling with iron nanoparticles is very important for biomedical applications [36]. Therefore, this study firstly investigated the applicability of IRDy800CW-MNPs in this field. The macrophage is commonly used as a cellular model to evaluate intravascularly administered agents, especially as it phagocytoses nanoparticles [10]. Therefore, this study employed the mouse macrophage RAW264.7 cell line to perform a cell-labeling assay. The cells were labeled with nanoparticles at various concentrations for 24 h. The cell labeling effect was evaluated by staining cells with Prussian blue and measuring the iron-loading of cells. The Prussian blue staining showed that the cells were effectively labeled by the MNPs and the IRDy800CW-MNPs (Figure 2). The blue-stained agglomerates of the iron nanoparticles in cells increased with the dose of nanoparticles in the cell culture media (Figure 2), which was in accordance with the results of the quantitative measurements of the relative iron-loading of cells using colorimetric and NIRF assays (Figure 3). In comparison, the NIRF assay reported the cellular iron-loading more sensitively than the normal colorimetric assay [69-72].

**Figure 4:**
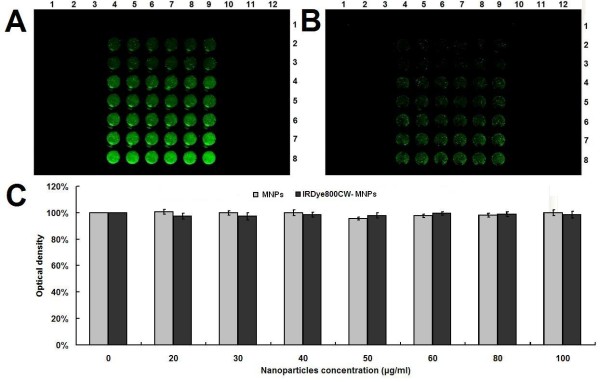
**Measurement of cell viability**. (A) NIRF signals of cells treated with MNPs (Column 1-3 and 10-12) and IRDy800CW-MNPs (Column 4-9) at doses of 0, 20, 30, 40, 50, 60, 80 and 100 μg/ml (From row 1-8) for 24 h. (B) NIRF signals of cells (A) after washing three times with PBS. (C) Quantitative measurement of cell viability by MTT assay. The error bars represent mean and standard deviations of experiments performed with 6 repeats.

The biocompatability of cells to the nanoparticles is also important to its applications. Therefore, we used an MTT assay to determine cell viability following treatment with the nanoparticles. The results revealed that the cell viability of RAW264.7 was not significantly (*p *> 0.05) affected by the various doses of both MNPs and IRDy800CW-MNPs (Figure 4). In comparison with MNPs, the IRDy800CW-labeling did not bring toxicity to the MNPs. These results demonstrate that the IRDy800CW-MNPs have increased biocompatability. A dose of 30 μg/ml of the nanoparticles used in this MTT assay corresponds to the optimal blood concentration of a nanoparticle imaging agent, Combidexe, which has been intravascularly administered in humans at 2.6 mg Fe/kg body weight [10,73].

### In vivo imaging with IRDy800CW-MNPs

Animal studies are indispensable to the clinical applications of nanoparticles. The biodistribution, metabolism, clearance and toxicity of nanoparticles must be examined in animal studies prior to their clinical application. In particular, these biological processes should be investigated in a dynamic and real-time form with living animals. In recent years, NIRF labeling has played an increasingly important role in *in vivo *studies [28-33]. Therefore, this study investigated the applicability of the IRDy800CW-MNPs in this field.

**Figure 5:**
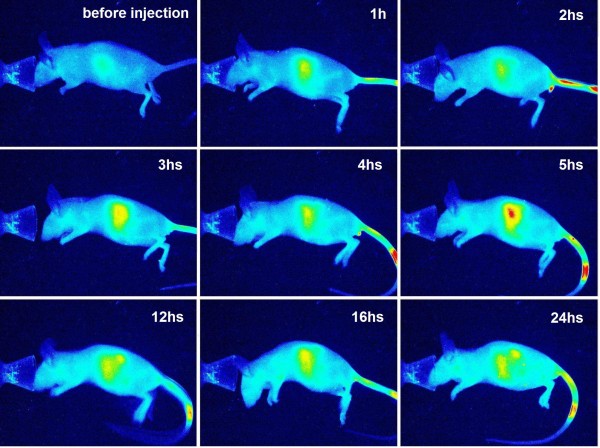
**NIRF imaging of a mouse administered IRDye800CW-MNPs at a dose of 2 mg/kg body weight**. The images are displayed in pseudo-color mode.

**Figure 6:**
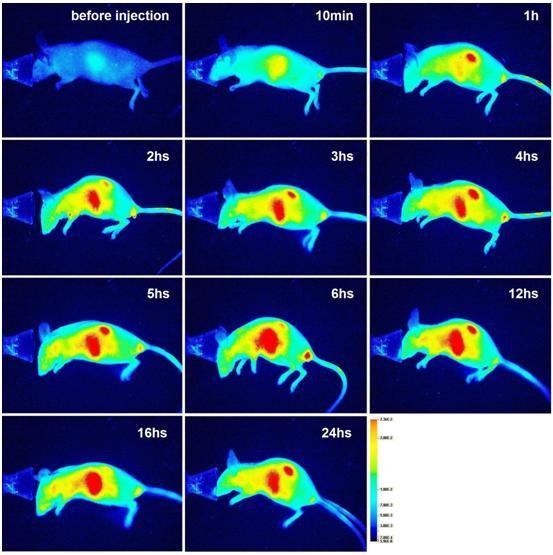
**NIRF imaging of a mouse administered the IRDy800CW-MNPs at a dose of 5 mg/kg body weight**. The images are displayed in pseudo-color mode.

**Figure 7:**
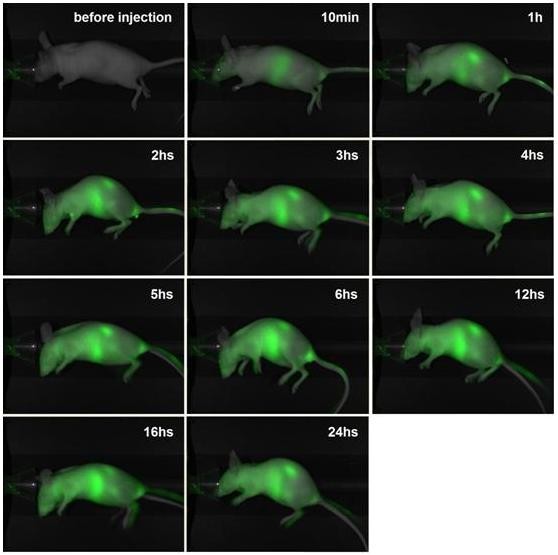
**NIRF imaging of a mouse administered the IRDye800CW-MNPs at a dose of 5 mg/kg body weight**. The images are displayed in an overlay mode of light channel image and NIRF channel image.

The *in vivo *studies were performed in a mouse model and employed a newly developed optical imaging instrument dedicated to small animal imaging, the Pearl Imager (LI-COR Biosciences) [74]. To obtain fine imaging effects, a naked mouse was used in this study. The mouse was first imaged prior to the administration of the nanoparticles to determine the value of the self-fluorescence background. Following this, the mouse was intravascularly administered IRDy800CW-MNPs at doses of 2 or 5 mg/kg body weight. The mouse was then discontinuously imaged at different time points. The real-time imaging of the mouse showed that the NIRF signal in the liver region and kidneys gradually intensified after injection of nanoparticles, reaching maximum levels at 6 h (Figure 5, 6 and 7), thereby demonstrating a gradual enrichment of the IRDy800CW-MNPs in these regions. Following this, the NIRF signal in these regions gradually decreased, revealing a gradual clearance of the IRDy800CW-MNPs. These results demonstrate that the whole dynamic process of biodistribution and clearance of MNPs in the mouse model could be monitored and tracked by the IRDy800CW labels and the small animal NIRF-imaging system, Pearl Image.

NIRF imaging of the mouse also clearly revealed that the intensity of signal in the liver region and kidneys was closely related to the dose of the intravenously injected IRDy800CW-MNPs. In comparison, the intensity of the NIRF signals in the liver and kidneys of the mouse injected with 2 mg/kg nanoparticles was much higher than that of the mouse injected with 5 mg/kg nanoparticles (Figure 5 and 6). This signal/dose relationship may be used to investigate the metabolism efficiency of the different doses of nanoparticles.

**Figure 8:**
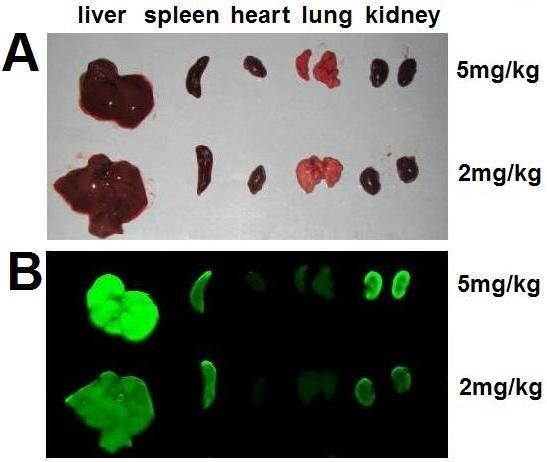
**NIRF imaging of organs of a IRDy800CW-MNPs-injected mouse**. (A) Light images of heart, lung, liver, spleen and kidney of the mouse administered the IRDye800CW-MNPs. (B) NIRF images of the same organs.

To clarify the exact biodistribution of nanoparticles in different organs, the mouse was sacrificed after imaging for 5 days, and the organs, including the heart, lungs, liver, spleen and kidneys were isolated and their NIRF signal was measured. The results revealed that the IRDy800CW-MNPs mainly distributed in the liver, spleen and kidneys (Figure 8), with minimal distribution in the heart and lungs. This agrees with the results of whole body imaging. It can be found that the intense NIRF signal in the liver region, as measured by live-body imaging, actually comes from two organs, the liver and spleen. The liver is the largest organ in the body of a mouse and the spleen is far smaller, but the spleen is closely attached to the liver; therefore, it cannot be discerned from the liver in the live-body imaging. However, the organ imaging clearly revealed its importance in evaluating the biodistribution of the nanoparticles. Taken together, the individual NIRF imaging of organs is an important supplement to live-body imaging, as it revealed that the *in vivo *biodistribution and clearance of the MNPs mainly related to these three organs.

In previous studies, it was found that the magnetic nanoparticles were mainly distributed in the liver and spleen [13,17,18,26,75-77]. This pattern of biodistribution is independent of the routes of administration, such as intravenous injection [13-15,18,53,75,78,79], intraperitoneal injection [26], intratracheally instillation [77], and inhalation [17]. These results are in agreement with our findings herein. The common highest distribution of various iron magnetic nanoparticles in liver and spleen closely relates to the reticulo-endothelial system (RES), also known as the mononuclear phagocytic system (MPS). The RES contains abundant phagocytic cells which can remove particulate materials from blood [80]. Therefore, the RES plays an important role in the biodistribution and clearance of nanoparticles *in vivo *[26,76,81,82]. Furthermore, the liver and spleen are the major RES organs in body, with Kupffer cells and macrophages being their main RES members, respectively. It was reported that over 75% of the magnetic nanoparticles were promptly sequestered by the RES, particularly by the liver [83]. It was also reported that after 6 h following administration, approximately 55% iron nanoparticles were enriched in the liver by the RES [76]. TEM observation of the liver and spleen revealed that Kupffer cells contained an increasing number of progressively larger phagolysosomes containing magnetic nanoparticles 7 days after injection, and the macrophages in the spleen contained magnetic nanoparticles in lysosomes [79]. It was also reported that the USPIO accumulated in macrophages of the liver, spleen, lymph nodes and bone marrow [14,73,84,85].

It was also reported that the magnetic nanoparticles were able to distribute in the kidneys, lungs, heart, brain, testes, uterus, ovary, bladder, thyroid, pancreas, and bone marrow [14]. However, the amount of nanoparticles distributed in these organs or tissues was far less than that in liver and spleen. This study revealed that the IRDy800CW-MNPs were also enriched in the kidneys. This may be related to the biological function of the kidneys, which is an important emunctory containing large a volume of blood undergoing filtration. The large difference in the NIRF intensity between the kidneys of mice injected with different doses of the IRDy800CW-MNPs (Figure 8) also demonstrated that the kidneys may play an important role in the biodistribution and clearance of iron nanoparticles.

The dose of the IRDy800CW-MNPs used in the *in vivo *imaging in this study is similar to those reported by other studies. The magnetic nanoparticles were reported to be intravascularly administered to mouse or rat at doses of 1 [15], 2 [15,83], 3 [86], 5 [15,87], and 10 mg Fe/kg body weight [76]. It was also reported that an intravascular nanoparticle imaging agent, Combidexe, was injected at 2.6 mg of Fe/kg body weight to humans for MRI [73].

This study did not measure physiological indexes and therefore cannot comment on any possible or potential effects of the IRDy800CW-MNPs to the health of mouse. However, careful observation of the mouse's behavior over the five days of *in vivo *imaging revealed that injection of the nanoparticles did not result in any observed adverse effects on activity, eating or drinking of the mouse. This implies that the IRDy800CW-MNPs may have better biocompatability to mice, which is the key small animal employed for the biomedical research of iron nanoparticles.

## Conclusion

This study manufactured water-soluble 12-nm DMSA-coated Fe_3_O_4 _nanoparticle labeled with a NIRF dye, IRDye800CW, and investigated its applicability in cell labeling and living body imaging. The results demonstrate that the IRDye800CW-labeled Fe_3_O_4 _nanoparticles effectively labeled a RAW264.7 cell, but did not significantly affect the cell viability. The animal studies demonstrate that the IRDye800CW-labeled Fe_3_O_4 _nanoparticles could sensitively and in real-time monitor the whole dynamic process of the biodistribution and clearance of the Fe_3_O_4 _nanoparticles in mouse. Therefore, IRDye800CW-labeled Fe_3_O_4 _nanoparticles provide a new selection of available iron nanoparticles labeled with NIRF dyes with an optimal wavelength for imaging centered at 800 nm, which can be applied to *in vitro *cell labeling and *in vivo *imaging.

## Methods

### Cells, animals and chemicals

The RAW264.7 cell line was purchased from the China Center for Type Culture Collection, Chinese Academy of Sciences (Shanghai, China). DMEM cell culture medium was purchased from Gibco, Invitrogen (CA, USA). The naked mouse (CByJ-Cg-Foxn1nu/J) was purchased from Model Animal Research Center of Nanjing, Nanjing University (Nanjing, China). The streptavidin-IRDye 800CW was purchased from Li-Cor Biosciences (Lincoln, NE, USA). The main chemicals, including EDC, HEPES, glutaraldehyde and paraformaldehyde, were purchased from Sigma Aldrich (MO, USA). Other chemicals, including potassium peroxydisulfate (K_2_S_2_O_8_), potassium ferrocyanide (KSCN), iron (III) chloride hexahydrate (FeCl_3_), and hydrochloric acid, were purchased from Sinopharm Chemical Reagent Co. Ltd (Shanghai, China).

### Preparation of IRDy800CW-MNPs

The water-soluble Fe_3_O_4 _nanoparticles were synthesized under the following conditions. Firstly, 2.7 g of FeCl_3_•6H_2_O was dissolved in 50 ml of methanol, followed by the addition of 8.5 ml oleic acid. Then, a solution with 1.2 g of NaOH in 100 ml methanol was dropwise added into the solution under magnetic stirring conditions. The observed brown precipitate was washed with methanol 4-5 times and dried under vacuum overnight to remove all solvents. The obtained waxy iron-oleate was dissolved in 1-octadecanol at 70°C and reserved as a stable stock solution at room temperature. One milliliter of the stock solution (0.39 mM) was mixed with 4 ml 1-octadecanol and 0.5 ml oleic acid. The reaction mixture was heated to 320°C at a constant heating rate of 3.3°C/min, in a nitrogen atmosphere, and maintained at that temperature for 30 min. The resulting solution was cooled and precipitated by an addition of excess ethanol and centrifugation. The precipitate containing Fe_3_O_4 _nanoparticles was washed 4-5 times with ethanol. To prepare water-soluble Fe_3_O_4 _nanoparticles, 100 mg of above Fe_3_O_4 _nanoparticles was dissolved in 10 ml chloroform, following which 50 μl triethylamine and 10 ml dimethyl sulfoxide (DMSO) containing 50 mg dispersed DMSA was added. The resulting solution was vortexed at 60°C for 12 h until a black precipitate was observed. The solution was subsequently centrifuged and the precipitate was carefully washed twice with ethanol and dissolved in 100 ml ethanol. To introduce more DMSA molecules onto the surface of Fe_3_O_4 _nanoparticles, 50 μl triethylamine was added to the above ethanol solution containing Fe_3_O_4 _nanoparticles, followed by the addition of a solution with 50 mg DMSA in 10 ml DMSO. The solution was again vortexed at 60°C for 12 h. The reaction solution was then centrifuged and the precipitate washed with ethanol 4-5 times carefully. The final MNPs were collected using a permanent magnet and transferred into 10 ml water.

The MNPs were labeled with NIR fluorophores by the following procedure. Six ml of nanoparticles (0.844 mg/ml Fe) were diluted into 24 ml and sonicated for 20 min. Following this, 10 mg EDC was added and sonicated for 40 min to activate the carboxyl groups on the surface of the nanoparticles. The solution was centrifuged at 12000 rpm for 10 min and the precipitate was resuspended in sterile deionized water. Then, 15 μl of Streptavidin-IRDye800CW was added to the resuspended nanoparticles and the nanoparticle solution was left on a rotator overnight. The nanoparticle solution was centrifuged at 12000 rpm for 10 min and the precipitate was washed 3 times with deionized water. Finally, the IRDye800CW-MNPs were resuspended in sterile deionized water.

The monodispersibility of MNPs and the IRDye800CW-MNPs was evaluated by TEM. Each of nanoparticles in the 30 μg/ml sample was added to a copper grid and observed with a JEM-2100 electron microscope (JEOL, Japan). The size of the nanoparticles was measured with Image Origin 6.1. The NIRF signal of the nanoparticles was detected with Odyssey Infrared Imaging System (Li-Cor). The fluorescent spectrum of the nanoparticles was measured using a Hitachi F-7000 Fluorescence Spectrophotometer. To verify that the IRDy800CW was covalently crosslinked to the nanoparticles and not by nonspecific absorption, the solution of NIRF-labeled nanoparticles was heated at 60°C for 15 min, and centrifuged to precipitate the nanoparticles. The supernatant and nanoparticles were collected separately, and the nanoparticles were resuspended in deionized water. The supernatants and the resuspended nanoparticles were detected with Odyssey Infrared Imaging System.

### Cell labeling with IRDy800CW-MNPs

To investigate the labeling of cells with nanoparticles, RAW264.7 cells were seeded into cell culture plates and cultured in DMEM supplemented with 10% fetal calf serum, penicillin (100 units/ml), streptomycin (100 μg/ml) and 10 mM HEPES in a humidified 5% CO_2 _atmosphere at 37°C for 24 h. Then, the culture medium was discarded and the cells were cultured with fresh media containing nanoparticles at different doses for 24 h.

The labeling effects were evaluated by staining cells with Prussian blue and measurement of the iron-loading of cells. Prussian blue staining, which stains iron nanoparticles blue, was performed as previously described [47-49,54-57]. The stained cells were observed with a light microscope (IX51, Olympus) and photographed using a Microscope Digital Camera (DP71, Olympus). The iron-loading of cells was measured with a common colorimetric assay, as previously described [69-72]. The iron-loading of the cells labeled by the IRDy800CW-MNPs was also evaluated by the NIRF signal. To measure the NIRF signal, cells were washed with PBS and lysed using SDS lysis buffer. The lysate was imaged and NIRF signal intensity was analyzed using the Odyssey Infrared Imaging System (Li-Cor).

The effect of labeling on the cell proliferation was evaluated by determining cell viability, which was assessed using a MTT assay, as reported elsewhere [82-84]. The cell viability of the nanoparticle-untreated cells (blank control) was defined as 100%, with the nanoparticle-treated cells being calculated as percentage of the control.

### In vivo imaging with IRDy800CW-MNPs

Before injection of the IRDy800CW-MNPs, the mouse was imaged on a newly-developed infrared fluorescence imaging system, Pearl Image (Li-Cor). Subsequently, the mouse was anesthetized by ether inhalation and the IRDy800CW-MNPs were administered to the mouse by intravascular injection via the tail vein, at doses of 2 or 5 mg Fe/kg body weight. The mouse was housed under normal conditions and discontinuously imaged with Pearl Image at various time points. Just before each image acquisition, the mouse was anesthetized by ether inhalation. All images were captured at the same exciting intensity. After housing and imaging for 5 days following the injection of nanoparticles, the mouse was sacrificed by an overdose of anesthesia and the organs, including the heart, lungs, liver, spleen and kidneys were immediately collected. The organs were washed with PBS and imaged with a normal camera and Odyssey Infrared Imaging System (Li-Cor).

## Abbreviations

MRI: Magnetic resonance imaging; NIRF: near infrared fluorescence; ICG: indocyanine green; PBS: Phosphate Buffer Solution; SDS: Sodium dodecyl sulfate; TEM: transmission electron microscopy; DMEM: Dulbecco's modified Eagle medium; CLIO: a form of MION (monocrystalline iron oxide nanoparticles) with cross-linked dextran coating: USPIO: dextran-coated SPIO (superparamagnetic iron oxide); EDC: 1-ethyl-3,(3-di-methylaminopropyl carbodiimide) hydrochloride; DMSA: *m*-2,3-dimercaptosuccinic acid; MTT: 3-(4,5-dimethylthiazol-2-yl)-2,5-diphenyl tetrazolium bromide

## Competing interests

The authors declare that they have no competing interests.

## Authors' contributions

YH, YL, ZC and NG synthesized nanoparticles; YL, YH and JW performed the cell labeling and the animal studies; YH, YL, ZC, NG and JW wrote the manuscript. All authors read and approved the final manuscript.

## References

[B1] Lewin M Carlesso N Tung CH Tang XW Cory D Scadden DT Weissleder R Tat peptide-derivatized magnetic nanoparticles allow in vivo tracking and recovery of progenitor cells Nat Biotechnol 2000 18 410 414 10.1038/74464 10748521

[B2] Nam JM Thaxton CS Mirkin CA Nanoparticle-based bio-bar codes for the ultrasensitive detection of proteins Science 2003 301 1884 1886 10.1126/science.1088755 14512622

[B3] Weissleder R Moore A Mahmood U Bhorade R Benveniste H Chiocca EA Basilion JP In vivo magnetic resonance imaging of transgene expression Nat Med 2000 6 351 355 10.1038/73219 10700241

[B4] Tiefenauer L Tuan Vo-Dinh Magnetic Nanoparticles as Contrast Agents for Medical Diagnosis Nanotechnology in Biology and Medicine 2007 Boca Raton: CRC Press 29/10

[B5] Ito A Shinkai M Honda H Kobayashi T Heat-inducible TNF-alpha gene therapy combined with hyperthermia using magnetic nanoparticles as a novel tumor-targeted therapy Cancer Gene Ther 2001 8 649 654 10.1038/sj.cgt.7700357 11593333

[B6] Hergt R Dutz S Magnetic particle hyperthermia-biophysical limitations of a visionary tumour therapy J Magn Magn Mater 2007 311 187 192 10.1016/j.jmmm.2006.10.1156

[B7] Lin BL Shen XD Cui S Application of nanosized Fe3O4 in anticancer drug carriers with target-orientation and sustained-release properties Biomed Mater 2007 2 132 134 10.1088/1748-6041/2/2/011 18458446

[B8] Shubayev VI Pisanic TR Jin S Magnetic nanoparticles for theragnostics Adv Drug Deliv Rev 2009 61 467 477 10.1016/j.addr.2009.03.007 19389434 PMC2700776

[B9] Pankhurst QA Connolly J Jones SK Dobson J Applications of magnetic nanoparticles in biomedicine J Phys D Appl Phys 2003 36 R167 R181 10.1088/0022-3727/36/13/201

[B10] Shaw SY Westly EC Pittet MJ Subramanian A Schreiber SL Weissleder R Perturbational profiling of nanomaterial biologic activity Proc Natl Acad Sci 2008 105 7387 7392 10.1073/pnas.0802878105 18492802 PMC2396702

[B11] Bacon BR Stark DD Park CH Saini S Groman EV Hahn PF Compton CC Ferrucci JT Jr Ferrite particles: a new magnetic resonance imaging contrast agent. Lack of acute or chronic hepatotoxicity after intravenous administration J Lab Clin Med 1987 110 164 171 3598345

[B12] Weissleder R Stark DD Engelstad BL Bacon BR Compton CC White DL Jacobs P Lewis J Superparamagnetic iron oxide: pharmacokinetics and toxicity AJR Am J Roentgenol 1989 152 167 173 2783272 10.2214/ajr.152.1.167

[B13] Lacava LM Lacava ZGM Azevedo RB Chaves SB Garcia VAP Silva O Pelegrini F Buske N Gansau C Da Silva MF Morais PC Use of magnetic resonance to study biodistribution of dextran-coated magnetic fluid intravenously administered in mice J Magn Magn Mater 2002 252 367 369 10.1016/S0304-8853(02)00654-6

[B14] Lacava LM Garcia VAP Kuckelhaus S Azevedo RB Lacava ZGM Silva O Pelegrini F Gansau C Buske N Morais PC Magnetic resonance and light microscopy investigation of a dextran coated magnetic fluid J Appl Phys 2003 93 7563 7565 10.1063/1.1540178

[B15] Briley-Saebo K Bjornerud A Grant D Ahlstrom H Berg T Kindberg GM Hepatic cellular distribution and degradation of iron oxide nanoparticles following single intravenous injection in rats: implications for magnetic resonance imaging Cell and Tissue Res 2004 316 315 323 10.1007/s00441-004-0884-8 15103550

[B16] Kalber TL Smith CJ Howe FA Griffiths JR Ryan AJ Waterton JC Robinson SP A longitudinal study of R2* and R2 magnetic resonance imaging relaxation rate measurements in murine liver after a single administration of 3 different iron oxide-based contrast agents Invest Radiol 2005 40 784 791 10.1097/01.rli.0000188025.66872.e4 16304482

[B17] Kwon JT Hwang SK Jin H Kim DS Minai-Tehrani A Yoon HJ Choi M Yoon TJ Han DY Kang YW Body distribution of inhaled fluorescent magnetic nanoparticles in the mice J Occup Health 2008 50 1 6 10.1539/joh.50.1 18285638

[B18] Lee PW Hsu SH Wang JJ Tsai JS Lin KJ Wey SP Chen FR Lai CH Yen TC Sung HW The characteristics, biodistribution, magnetic resonance imaging and biodegradability of superparamagnetic core-shell nanoparticles Biomaterials 2010 31 1316 1324 10.1016/j.biomaterials.2009.11.010 19959224

[B19] Hatanaka S Matsushita N Abe M Nishimura K Hasegawa M Handa H Direct immobilization of fluorescent dyes onto ferrite nanoparticles during their synthesis from aqueous solution J Appl Phys 2003 93 7569 7570 10.1063/1.1558677

[B20] Yoon TJ Kim JS Kim BG Yu KN Cho MH Lee JK Multifunctional nanoparticles possessing a "magnetic motor effect" for drug or gene delivery Angew Chem Int Ed Engl 2005 44 1068 1071 10.1002/anie.200461910 15635729

[B21] Guo J Yang W Deng Y Wang C Fu S Organic-dye-coupled magnetic nanoparticles encaged inside thermoresponsive PNIPAM Microcapsules Small 2005 1 737 743 10.1002/smll.200400145 17193517

[B22] Kwon JT Kim DS Minai-Tehrani A Hwang SK Chang SH Lee ES Xu CX Lim HT Kim JE Yoon BI Inhaled fluorescent magnetic nanoparticles induced extramedullary hematopoiesis in the spleen of mice J Occup Health 2009 51 423 431 10.1539/joh.L8159 19706996

[B23] Bertorelle F Wilhelm C Roger J Gazeau F Menager C Cabuil V Fluorescence-modified superparamagnetic nanoparticles: intracellular uptake and use in cellular imaging Langmuir 2006 22 5385 5391 10.1021/la052710u 16732667

[B24] Laemmel E Genet M Le Goualher G Perchant A Le Gargasson JF Vicaut E Fibered confocal fluorescence microscopy (Cell-viZio) facilitates extended imaging in the field of microcirculation. A comparison with intravital microscopy J Vasc Res 2004 41 400 411 10.1159/000081209 15467299

[B25] Lim YT Kim S Nakayama A Stott NE Bawendi MG Frangioni JV Selection of quantum dot wavelengths for biomedical assays and imaging Mol Imaging 2003 2 50 64 10.1162/153535003765276282 12926237

[B26] Kim JS Yoon TJ Yu KN Kim BG Park SJ Kim HW Lee KH Park SB Lee JK Cho MH Toxicity and tissue distribution of magnetic nanoparticles in mice Toxicol Sci 2006 89 338 347 10.1093/toxsci/kfj027 16237191

[B27] Frangioni JV In vivo near-infrared fluorescence imaging Curr Opin Chem Biol 2003 7 626 634 10.1016/j.cbpa.2003.08.007 14580568

[B28] Achilefu S Lighting up tumors with receptor-specific optical molecular probes Technol Cancer Res Treat 2004 3 393 409 15270591 10.1177/153303460400300410

[B29] Ntziachristos V Bremer C Weissleder R Fluorescence imaging with near-infrared light: new technological advances that enable in vivo molecular imaging Eur Radiol 2003 13 195 208 12541130 10.1007/s00330-002-1524-x

[B30] Weissleder R A clearer vision for in vivo imaging Nat Biotechnol 2001 19 316 317 10.1038/86684 11283581

[B31] Loo C Lowery A Halas N West J Drezek R Immunotargeted nanoshells for integrated cancer imaging and therapy Nano Lett 2005 5 709 711 10.1021/nl050127s 15826113

[B32] Sevick-Muraca EM Houston JP Gurfinkel M Fluorescence-enhanced, near infrared diagnostic imaging with contrast agents Curr Opin Chem Biol 2002 6 642 650 10.1016/S1367-5931(02)00356-3 12413549

[B33] Weissleder R Ntziachristos V Shedding light onto live molecular targets Nat Med 2003 9 123 128 10.1038/nm0103-123 12514725

[B34] Kumar R Roy I Ohulchanskky TY Vathy LA Bergey EJ Sajjad M Prasad PN In Vivo Biodistribution and Clearance Studies Using Multimodal Organically Modified Silica Nanoparticles ACS Nano 2010 4 699 708 10.1021/nn901146y 20088598 PMC2827663

[B35] Altinoglu EI Russin TJ Kaiser JM Barth BM Eklund PC Kester M Adair JH Near-infrared emitting fluorophore-doped calcium phosphate nanoparticles for in vivo imaging of human breast cancer ACS Nano 2008 2 2075 2084 10.1021/nn800448r 19206454

[B36] Maxwell DJ Bonde J Hess DA Hohm SA Lahey R Zhou P Creer MH Piwnica-Worms D Nolta JA Fluorophore-conjugated iron oxide nanoparticle labeling and analysis of engrafting human hematopoietic stem cells Stem Cells 2008 26 517 524 10.1634/stemcells.2007-0016 18055451 PMC2863008

[B37] McCann CM Waterman P Figueiredo JL Aikawa E Weissleder R Chen JW Combined magnetic resonance and fluorescence imaging of the living mouse brain reveals glioma response to chemotherapy Neuroimage 2009 45 360 369 10.1016/j.neuroimage.2008.12.022 19154791 PMC2707831

[B38] Josephson L Kircher MF Mahmood U Tang Y Weissleder R Near-infrared fluorescent nanoparticles as combined MR/optical imaging probes Bioconjug Chem 2002 13 554 560 10.1021/bc015555d 12009946

[B39] Jaffer FA Nahrendorf M Sosnovik D Kelly KA Aikawa E Weissleder R Cellular imaging of inflammation in atherosclerosis using magnetofluorescent nanomaterials Mol Imaging 2006 5 85 92 16954022

[B40] Montet X Ntziachristos V Grimm J Weissleder R Tomographic fluorescence mapping of tumor targets Cancer Res 2005 65 6330 6336 10.1158/0008-5472.CAN-05-0382 16024635

[B41] Jaffer FA Sosnovik DE Nahrendorf M Weissleder R Molecular imaging of myocardial infarction J Mol Cell Cardiol 2006 41 921 933 10.1016/j.yjmcc.2006.09.008 17067633

[B42] Funovics M Montet X Reynolds F Weissleder R Josephson L Nanoparticles for the optical imaging of tumor E-selectin Neoplasia 2005 7 904 911 10.1593/neo.05352 16242073

[B43] Chen ZP Zhang Y Zhang S Xia JG Liu JW Xu K Gu N Preparation and characterization of water-soluble monodisperse magnetic iron oxide nanoparticles via surface double-exchange with DMSA Colloid Surf A 2008 316 210 216 10.1016/j.colsurfa.2007.09.017

[B44] Kalambur VS Longmire EK Bischof JC Cellular level loading and heating of superparamagnetic iron oxide nanoparticles Langmuir 2007 23 12329 12336 10.1021/la701100r 17960940

[B45] Rad AM Janic B Iskander A Soltanian-Zadeh H Arbab AS Measurement of quantity of iron in magnetically labeled cells: comparison among different UV/VIS spectrometric methods Biotechniques 2007 43 627 628 630, 632 passim 10.2144/000112599 18072592

[B46] Hafelli UO Riffle JS Harris-Shekhawat L Carmichael-Baranauskas A Mark F Dailey JP Bardenstein D Cell Uptake and in Vitro Toxicity of Magnetic Nanoparticles Suitable for Drug Delivery Mol Pharm 2009 6 1417 1428 10.1021/mp900083m 19445482

[B47] Auffan M Decome L Rose J Orsiere T De Meo M Briois V Chaneac C Olivi L Berge-Lefranc JL Botta A In vitro interactions between DMSA-coated maghemite nanoparticles and human fibroblasts: A physicochemical and cyto-genotoxical study Environ Sci Technol 2006 40 4367 4373 10.1021/es060691k 16903272

[B48] Ju SH Teng GJ Zhang Y Ma M Chen F Ni YC In vitro labeling and MRI of mesenchymal stem cells from human umbilical cord blood Magn Reson Imaging 2006 24 611 617 10.1016/j.mri.2005.12.017 16735183

[B49] Song M Moon WK Kim Y Lim D Song IC Yoon BW Labeling efficacy of superparamagnetic iron oxide nanoparticles to human neural stem cells: Comparison of ferumoxides, monocrystalline iron oxide, cross-linked iron oxide (CLIO)-NH2 and tat-CLIO Korean J Radiol 2007 8 365 371 10.3348/kjr.2007.8.5.365 17923778 PMC2626816

[B50] Nakajima N Ikada Y Mechanism of Amide Formation by Carbodiimide for Bioconjugation in Aqueous-Media Bioconjugate Chem 1995 6 123 130 10.1021/bc00031a015 7711098

[B51] Fauconnier N Pons JN Roger J Bee A Thiolation of maghemite nanoparticles by dimercaptosuccinic acid J Colloid Interface Sci 1997 194 427 433 10.1006/jcis.1997.5125 9398425

[B52] Valois CRA Braz JM Nunes ES Vinolo MAR Lima ECD Curi R Kuebler WM Azevedo RB The effect of DMSA-functionalized magnetic nanoparticles on transendothelial migration of monocytes in the murine lung via a beta(2) integrin-dependent pathway Biomaterials 2010 31 366 374 10.1016/j.biomaterials.2009.09.053 19822361

[B53] Chaves SB Lacava LM Lacava ZGM Silva O Pelegrini F Buske N Gansau C Morais PC Azevedo RB Light microscopy and magnetic resonance characterization of a DMSA-coated magnetic fluid in mice IEEE T MAGN 2002 38 3231 3233 10.1109/TMAG.2002.802495

[B54] Frank JA Miller BR Arbab AS Zywicke HA Jordan EK Lewis BK Bryant LH Bulte JWM Clinically applicable labeling of mammalian and stem cells by combining; Superparamagnetic iron oxides and transfection agents Radiology 2003 228 480 487 10.1148/radiol.2281020638 12819345

[B55] Riviere C Boudghene FP Gazeau F Roger J Pons JN Laissy JP Allaire E Michel JB Letourneur D Deux JF Iron oxide nanoparticle-labeled rat smooth muscle cells: Cardiac MR imaging for cell graft monitoring and quantitation Radiology 2005 235 959 967 10.1148/radiol.2353032057 15845788

[B56] Wilhelm C Billotey C Roger J Pons JN Bacri JC Gazeau F Intracellular uptake of anionic superparamagnetic nanoparticles as a function of their surface coating Biomaterials 2003 24 1001 1011 10.1016/S0142-9612(02)00440-4 12504522

[B57] Wilhelm C Gazeau F Universal cell labelling with anionic magnetic nanoparticles Biomaterials 2008 29 3161 3174 10.1016/j.biomaterials.2008.04.016 18455232

[B58] Wilhelm C Gazeau F Bacri JC Magnetophoresis and ferromagnetic resonance of magnetically labeled cells Eur Biophys J 2002 31 118 125 10.1007/s00249-001-0200-4 12012115

[B59] Wilhelm C Gazeau F Roger J Pons JN Bacri JC Interaction of anionic superparamagnetic nanoparticles with cells: Kinetic analyses of membrane adsorption and subsequent internalization Langmuir 2002 18 8148 8155 10.1021/la0257337

[B60] Marshall MV Draney D Sevick-Muraca EM Olive DM Single-Dose Intravenous Toxicity Study of IRDye 800CW in Sprague-Dawley Rats Mol Imaging Biol 2010 10.1007/s11307-010-0317-x PMC2978892 20376568

[B61] Blum G von Degenfeld G Merchant MJ Blau HM Bogyo M Noninvasive optical imaging of cysteine protease activity using fluorescently quenched activity-based probes Nat Chem Biol 2007 3 668 677 10.1038/nchembio.2007.26 17828252

[B62] Adams KE Ke S Kwon S Liang F Fan Z Lu Y Hirschi K Mawad ME Barry MA Sevick-Muraca EM Comparison of visible and near-infrared wavelength-excitable fluorescent dyes for molecular imaging of cancer J Biomed Opt 2007 12 024017 10.1117/1.2717137 17477732

[B63] Tanaka E Ohnishi S Laurence RG Choi HS Humblet V Frangioni JV Real-time intraoperative ureteral guidance using invisible near-infrared fluorescence J Urology 2007 178 2197 2202 10.1016/j.juro.2007.06.049 PMC2505174 17870110

[B64] Foster AE Kwon S Ke S Lu A Eldin K Sevick-Muraca E Rooney CM In vivo fluorescent optical imaging of cytotoxic T lymphocyte migration using IRDye800CW near-infrared dye Applied Optics 2008 47 5944 5952 10.1364/AO.47.005944 19122737 PMC2744150

[B65] Kovar JL Volcheck W Sevick-Muraca E Simpson MA Olive DM Characterization and performance of a near-infrared 2-deoxyglucose optical imaging agent for mouse cancer models Anal Biochem 2009 384 254 262 10.1016/j.ab.2008.09.050 18938129 PMC2720560

[B66] Wang GJ Liu Y Qin A Shah SV Deng ZB Xiang X Cheng Z Liu C Wang J Zhang L Thymus exosomes-like particles induce regulatory T cells J Immunol 2008 181 5242 5248 18832678 10.4049/jimmunol.181.8.5242 PMC4319673

[B67] Duysen EG Lockridge O Whole body and tissue imaging of the butyrylcholinesterase knockout mouse injected with near infrared dye labeled butyrylcholinesterase Chem-Biol Interact 2008 175 119 124 10.1016/j.cbi.2008.02.011 18486120

[B68] Tanaka E Choi HS Fujii H Bawendi MG Frangioni JV Image-guided oncologic surgery using invisible light: Completed pre-clinical development for sentinel lymph node mapping Ann Surg Oncol 2006 13 1671 1681 10.1245/s10434-006-9194-6 17009138 PMC2474791

[B69] Tucker BA Rahirntula M Mearow KM A procedure for selecting and culturing subpopulations of neurons from rat dorsal root ganglia using magnetic beads Brain Res Protoc 2005 16 50 57 10.1016/j.brainresprot.2005.10.004 16309950

[B70] Cheng FY Su CH Yang YS Yeh CS Tsai CY Wu CL Wu MT Shieh DB Characterization of aqueous dispersions of Fe3O4 nanoparticles and their biomedical applications Biomaterials 2005 26 729 738 10.1016/j.biomaterials.2004.03.016 15350777

[B71] Liu SY Long L Yuan Z Yin LP Liu R Effect and intracellular uptake of pure magnetic Fe3O4 nanoparticles in the cells and organs of lung and liver Chinese Med J 2009 122 1821 1825 19781333

[B72] Jain TK Morales MA Sahoo SK Leslie-Pelecky DL Labhasetwar V Iron oxide nanoparticles for sustained delivery of anticancer agents Mol Pharm 2005 2 194 205 10.1021/mp0500014 15934780

[B73] Harisinghani MG Saini S Hahn PF Weissleder R Mueller PR MR imaging of lymph nodes in patients with primary abdominal and pelvic malignancies using ultrasmall superparamagnetic iron oxide (Combidex) Acad Radiol 1998 5 S167 S169 10.1016/S1076-6332(98)80095-0 9561072

[B74] Chen Y Dhara S Banerjee SR Byun Y Pullambhatla M Mease RC Pomper MG A low molecular weight PSMA-based fluorescent imaging agent for cancer Biochem Biophys Res Commun 2009 390 624 629 10.1016/j.bbrc.2009.10.017 19818734 PMC2787846

[B75] Gamarra LF Pontuschka WM Amaro E Costa-Filho AJ Brito GES Vieira ED Carneiro SM Escriba DM Falleiros AMF Salvador VL Kinetics of elimination and distribution in blood and liver of biocompatible ferrofluids based on Fe3O4 nanoparticles: An EPR and XRF study Mat Sci Eng C-Bio S 2008 28 519 525 10.1016/j.msec.2007.06.005

[B76] Jain TK Reddy MK Morales MA Leslie-Pelecky DL Labhasetwar V Biodistribution, clearance, and biocompatibility of iron oxide magnetic nanoparticles in rats Mol Pharm 2008 5 316 327 10.1021/mp7001285 18217714

[B77] Zhu MT Feng WY Wang Y Wang B Wang M Ouyang H Zhao YL Chai ZF Particokinetics and extrapulmonary translocation of intratracheally instilled ferric oxide nanoparticles in rats and the potential health risk assessment Toxicol Sci 2009 107 342 351 10.1093/toxsci/kfn245 19023088

[B78] Lacava LM Garcia VAP Kuckelhaus S Azevedo RB Sadeghiani N Buske N Morais PC Lacava ZGM Long-term retention of dextran-coated magnetite nanoparticles in the liver and spleen J Magn Magn Mater 2004 272-76 2434 2435 10.1016/j.jmmm.2003.12.852

[B79] Lacava LM Azevedo RB Bao SN Morais PC Lacava ZGM Dextran-coated magnetite nanoparticles effects in mice: a transmission electron microscopy investigation Magnetics Conference, 2005 INTERMAG Asia 2005 Digests of the IEEE International 2005 455 456 full_text

[B80] Ferrucci JT Stark DD Iron oxide-enhanced MR imaging of the liver and spleen: review of the first 5 years AJR Am J Roentgenol 1990 155 943 950 2120963 10.2214/ajr.155.5.2120963

[B81] Gajdosikova A Gajdosik A Koneracka M Zavisova V Stvrtina S Krchnarova V Kopcansky P Tomasovicova N Stolc S Timko M Acute toxicity of magnetic nanoparticles in mice Neuro Endocrinol 2006 27 96 99 17159789

[B82] Zhai Y Wang XL Wang XM Xie H Gu HC Acute toxicity and irritation magnetic of water-based dextran-coated magnetic fluid injected in mice J Biomed Mater Res 2008 85A 582 587 10.1002/jbm.a.31189 17806122

[B83] Chouly C Pouliquen D Lucet I Jeune JJ Jallet P Development of superparamagnetic nanoparticles for MRI: Effect of particle size, charge and surface nature on biodistribution J Microencapsul 1996 13 245 255 10.3109/02652049609026013 8860681

[B84] Weissleder R Heautot JF Schaffer BK Nossiff N Papisov MI Bogdanov A Brady TJ Mr Lymphography - Study of a High-Efficiency Lymphotrophic Agent Radiology 1994 191 225 230 8134576 10.1148/radiology.191.1.8134576

[B85] Anzai Y Mclachlan S Morris M Saxton R Lufkin RB Dextran-Coated Superparamagnetic Iron-Oxide, an Mr Contrast Agent for Assessing Lymph-Nodes in the Head and Neck AJNR Am J Neuroradiol 1994 15 87 94 7511324 PMC8332075

[B86] Okon E Pouliquen D Okon P Kovaleva ZV Stepanova TP Lavit SG Kudryavtsev BN Jallet P Biodegradation of Magnetite Dextran Nanoparticles in the Rat - a Histologic and Biophysical Study Lab Invest 1994 71 895 903 7807971

[B87] Briley-Saebo K Hustvedt SA Haldorsen A Bjornerud A Long-term imaging effects in rat liver after a single injection of an iron oxide nanoparticle based MR contrast agent J Magn Reson Imaging 2004 20 622 631 10.1002/jmri.20175 15390223

